# Hip fracture rate and osteoporosis treatment in Ontario: A population-based retrospective cohort study

**DOI:** 10.1007/s11657-024-01402-6

**Published:** 2024-06-25

**Authors:** Hajar AbuAlrob, George Ioannidis, Susan Jaglal, Andrew Costa, Lauren E. Grifith, Lehana Thabane, Jonathan D. Adachi, Cathy Cameron, Loretta Hillier, Arthur Lau, Alexandra Papaioannou

**Affiliations:** 1 Department of Health Research Methods, Evidence, and Impact, McMaster University, Hamilton, Canada; 2 Department of Medicine, McMaster University, Hamilton, Canada; 3 Department of Physical Therapy, University of Toronto, Toronto, Canada; 4 Department of Pediatrics and Anesthesia, McMaster University, Hamilton, Canada; 5 Institute for Clinical Evaluative Sciences, Toronto, Canada; 6 Geras Centre for Aging Research, Hamilton, Canada

**Keywords:** Long-term care, Osteoporosis, Fragility fracture, Real-world data, Post-fracture care

## Abstract

***Summary*:**

This population-based study analyzes hip fracture and osteoporosis treatment rates among older adults, stratified by place of residence prior to fracture. Hip fracture rates were higher among older adults living in the community and discharged to long-term care (LTC) after fracture, compared to LTC residents and older adults living in the community. Only 23% of LTC residents at high fracture risk received osteoporosis treatment.

**Purpose:**

This population-based study examines hip fracture rate and osteoporosis management among long-term care (LTC) residents > 65 years of age compared to community-dwelling older adults at the time of fracture and admitted to LTC after fracture, in Ontario, Canada.

**Methods:**

Healthcare utilization and administrative databases were linked using unique, encoded identifiers from the ICES Data Repository to estimate hip fractures (identified using the Public Health Agency of Canada algorithm and International Classification of Diseases (ICD)-10 codes) and osteoporosis management (pharmacotherapy) among adults > 66 years from April 1, 2014 to March 31, 2018. Sex-specific and age-standardized rates were compared by pre-fracture residency and discharge location (i.e., LTC to LTC, community to LTC, or community to community). Fracture risk was determined using the Fracture Risk Scale (FRS).

**Results:**

At baseline (2014/15), the overall age-standardized hip fracture rate among LTC residents was 223 per 10,000 person-years (173 per 10,000 females and 157 per 10,000 males), 509 per 10,000 person-years (468 per 10,000 females and 320 per 10,000 males) among the community to LTC cohort, and 31.5 per 10,000 person-years (43.1 per 10,000 females and 25.6 per 10,000 males). During the 5-year observation period, the overall annual average percent change (APC) for hip fracture increased significantly in LTC (AAPC =  + 8.6 (95% CI 5.0 to 12.3; *p* = 0.004) compared to the community to LTC group (AAPC =  + 2.5 (95% CI − 3.0 to 8.2; *p* = 0.248)) and the community-to-community cohort (AAPC − 3.8 (95% CI − 6.7 to − 0.7; *p* = 030)). However, hip fracture rate remained higher in the community to LTC group over the study period. There were 33,594 LTC residents identified as high risk of fracture (FRS score 4 +), of which 7777 were on treatment (23.3%).

**Conclusion:**

Overall, hip fracture rates have increased in LTC and among community-dwelling adults admitted to LTC after fracture. However, hip fracture rates among community-dwelling adults have decreased over time. A non-significant increase in osteoporosis treatment rates was observed among LTC residents at high risk of fracture (FRS4 +). Residents in LTC are at very high risk for fracture and require individualized based on goals of care and life expectancy.

**Supplementary Information:**

The online version contains supplementary material available at 10.1007/s11657-024-01402-6.

## Introduction

Hip fractures in long-term care (LTC) are the most common type of fracture (49% of all fractures) [1] and are twice the rate of those living in the community [1, 2]. It is estimated that 45% of LTC residents with a hip fracture in Canada die within 12 months [3] and of the survivors, 48% are no longer ambulatory [3–5]. LTC residents may be at higher risk of fracture due to the characteristics of the LTC cohort. For example, older adults in LTC are typically older, frailer, at increased risk of falls and fractures, and more likely to experience mobility limitation compared to community-dwelling seniors [1, 2]. Thus, osteoporotic fractures and treatment rates may vary among older adults in different settings (i.e., community vs. LTC).

Hip fracture prevention in LTC is often complicated by the medical complexity of the LTC resident and difficulty in determining fracture risk. Fracture risk assessment tools, including the Canadian Association of Radiologists and Osteoporosis Canada tool (CAROC) and the Canadian Fracture Risk Assessment Tool (FRAX) are not generalizable for LTC as they typically provide a 10-year fracture risk assessment timeframe (however, the mean life expectance of LTC resident is 2.4 years) [6, 7]. Additionally, these fracture risk assessment tools do not include LTC-specific risk factors such as wandering, cognitive impairment and transfer status, for predicting new hip fractures and were not developed for the frail, institutionalized LTC residents, making them unsuitable for decision making in LTC [8–10].

Although a prior fragility fracture is a well-established risk factor for future fracture, many studies suggest that the increased risk of a subsequent fracture is particularly acute immediately after an index fracture and wanes progressively over the next 2 years (i.e., imminent risk) [11]. Although fracture risk assessment tools such as FRAX predicts the 10-year fracture risk, some studies have suggested that this underestimates the imminent fracture risk. However, recent evidence has shown that FRAX could be adapted to predict fracture over a shorter period [12, 13].

The Fracture Risk Scale (FRS) is a validated tool that predicts LTC residents at risk for fracture within 1 year [7]. It uses data readily available in the Resident Assessment Instrument Minimum Data Set (RAI-MDS 2.0), which is a comprehensive, standardized assessment that gathers a wide range of socio-demographic and clinical characteristics on admission and a quarterly basis thereafter. The FRS was developed using Ontario residents’ data from the RAI-MDS 2.0, the Discharge Abstract Database (DAD) and the National Ambulatory Care Reporting System (NACRS) and has been externally validated across several Canadian provinces [14]. The FRS can be easily implemented in LTC as it can be obtained from the interRAI-Long Term Care Facility assessment. Implemented in 2018, the FRS is a new tool that allows objective data on treatment rates based on risk factors, to be obtained and can provide new and valuable insight into the effectiveness of treatment strategies within the LTC setting.

This study aims to compare hip fracture rates in older adults living either in LTC or the community at the time of fracture, examine osteoporosis treatment rates in LTC and across fracture risk levels, and compare the differences in demographic and temporal trends between 2014 and 2018. We hypothesize that fracture and treatment rate will vary among older adults depending on place of residence prior to fracture.

## Methods

### Study design

This population-based retrospective cohort study that used de-identified health administrative data from the publicly funded healthcare system in Ontario. Data for this study was obtained from ICES (formerly named the Institute for Clinical Evaluative Sciences), an independent, non-profit research institute funded by an annual grant from the Ontario Ministry of Health (MOH) and the Ministry of Long-Term Care (MLTC). As a prescribed entity under Ontario’s privacy legislation, ICES is authorized to collect and use health care data for the purposes of health system analysis, evaluation, and decision support. Secure access to these data is governed by policies and procedures that are approved by the Information and Privacy Commissioner of Ontario. Administrative datasets from the ICES Data Repository were linked using encrypted patient-specific identifiers (ICES-specific key number (IKN)) and analyzed at ICES [15] health services records for as many as 13 million residents living in Ontario were included [15].

### Study participants

All Ontario individuals > 65 years old, alive on the first day of the fiscal year of interest (FY April 1, 2014/15 to March 31, 2018/19) and who had a hip fracture related to osteoporosis were identified from healthcare records. Individuals aged > 65 years were chosen to ensure that participants had at least one full year of eligibility for public drug coverage (i.e., Ontario Drug Benefit (ODB) program) prior to index fracture. An individual was classified as living in a LTC home during a given year if they received a visit from a physician to a LTC home, filled a prescription while living in LTC, or had been admitted to and not yet discharged from LTC. LTC status was identified from the ODB, OHIP, and Continuing Care Reporting System (for Chronic Care) CCRS-LTC databases. The CCRS provides demographic and clinical information for all individuals residing in Ontario’s publicly funded LTC facilities. The ODB database provides physician billing claims and prescription drug data on all adults aged 65 + years, while the OHIP database provides physician billing claims from Nursing Homes or a Home for the Aged and Non-emergency LTC inpatient services (with fee code). The Master Numbering System database (MNS) was used to differentiate between chronic care and LTC residents. See Supplementary information Table 1 for a list of databases. Patients were excluded from the cohort if they had missing sex, were aged > 105 years old, missing or invalid Local Health Integration Network (LHIN) data, were not eligible during at least one quarter in the calendar year of interest or had died prior to the first day of the index year. LHIN’s (now operating under the name Home and Community Care Support Services) are regional health authorities in Ontario, Canada, responsible for planning, integrating, and funding health services at the local level [16]. Their primary focus includes service delivery for home care, LTC home placement services, and facilitation of access to community resources and supports. In this study, data was stratified by LHIN’s to examine if there are any geographic differences among outcomes accordingly.

**Table 1:** Overall Age-Standardized Hip fracture rates per 10,000 person-years among cohorts

	LTC-LTC	Community-LTC	Community-Community
Year	Overall	Overall	Overall
2014–2015	223.6 (212.3–235.4)	509.2 (479.9–539.8)	31.5 (30.6–32.4)
2015–2016	241.7 (229.7–254.0)	481.3 (453.1–510.8)	28.4 (27.6–29.2)
2016–2017	249.1 (236.6–262.1)	477.9 (448.8–508.4)	27.8 (27.1–28.6)
2017–2018	277.7 (263.7–292.3)	534.0 (501.8–567.8)	26.8 (26.1–27.5)
2018–2019	315.7 (299.4–332.7)	549.4 (514.9–585.6)	26.7 (26.0–27.4)
Absolute change	92.1	40.2	− 4.8
Relative change %	41.2%	7.9%	− 15.2%

^*^Data is presented as rate per 10,000 person-years (confidence interval)

*“Source: ICES AHRQ Project 2022 0900 819 002”*

For fracture risk assessment and osteoporosis treatment rate, those reported on the RAI-MDS 2.0 to have end-stage disease (defined as 6 months or less to live), in comatose (those in a state of deep unarousable unconsciousness unable to respond to external stimuli), receiving hospice care (is identified as being in a program for terminally ill persons where services are necessary for the palliation and management of terminal illness and related condition), individuals with an expected short LTC stay (< 90-day admission), or individuals with missing data to determine their fracture risk score were excluded. The sample included 627 LTC facilities across the province of Ontario.

Clinical LTC data was obtained from the Resident Assessment Instrument Minimum Data Set Version 2.0 (RAI-MDS 2.0). The RAI-MDS is a valid and reliable standardized assessment that is completed upon admission to LTC and quarterly thereafter for all residents [17]. RAI-MDS is completed by the resident’s care team who gather information from the resident, their family members and health care providers, and medical records. It includes health and social assessment forms that allow a clinician to provide a comprehensive assessment of the quality of care provided to the resident and identify the need for further evaluations of specific conditions or the need for individualized care [18].

Data from the RAI-MDS was linked to the Discharge Abstract Database (DAD) and National Ambulatory Care Reporting System (NACRS) to capture incident hip fracture and determine the residents’ FRS. The DAD captures inpatient acute care hospital stay data while the NACRS captures emergency department visits [19, 20]. Physician billing records were also searched for evidence of fractures not found in the hospital-based data; the algorithm required at least two physician claims dated within 91 days of one another. Individuals believed to have suffered a hip fracture related to osteoporosis were identified using the Public Health Agency of Canada algorithm (PHAC), using data sources from the National Ambulatory Care Reporting System (NACRS), Discharge Abstract Database (DAD), Same Day Surgery (SDS), National Rehabilitation Reporting System (NRS), Ontario Health Insurance Plan Claims Database (OHIP), and Continuing Care Reporting System (CCRS) [21]. Using International Classification of Disease 10 codes (ICD-10), LTC residents were coded as having a hip fracture [hip (S72.0, S72.1 S72.2) within 1-year of their admission assessments. See Supplementary Table 2. The Ontario Drug Benefit (ODB) database includes data on prescription medication claims (i.e., drug identifier, quantity, number of days supplied, date dispensed) for individuals covered under the provincial drug program including adults 65 years and older and LTC residents. Drug identification numbers (DIN’s) were used to identify osteoporosis medications (i.e., bisphosphonates (i.e., Risedronate, Alendronate, Zoledronic Acid), denosumab, raloxifene licensed for use to treat osteoporosis in Canada) as outlined by the PHAC. Over-the-counter supplements such as calcium and vitamin D were not assessed as these data are not available in the administrative datasets of the ICES Data Repository. A look-back of 1 year was used to pull osteoporosis medication records. Participants’ prescription for osteoporosis medication was identified if they obtained a new prescription at any point during a specific fiscal year. The initial prescription filled during the year was termed the ‘index prescription,’ considered ‘new’ if the individual had not filled a prescription for any osteoporosis medication in the year preceding the index date. Individuals were then tracked for 1 year following the index date to determine if they continued to fill prescriptions during the subsequent year. The number of days of drug supplied on each prescription was used to determine when it should be refilled (ex. For denosumab days = 180). After the first prescription for each individual runs out (based on the days of medication), there is a grace period of 60 days to refill prescription, if the prescription is not filled until the end of the year, then the individual is not considered to be taking the drug 1 year later. The use of the data in this project is authorized under Sect. 45 of Ontario’s Personal Health Information Protection Act (PHIPA).

### Outcome definition

**Fig. 1:**
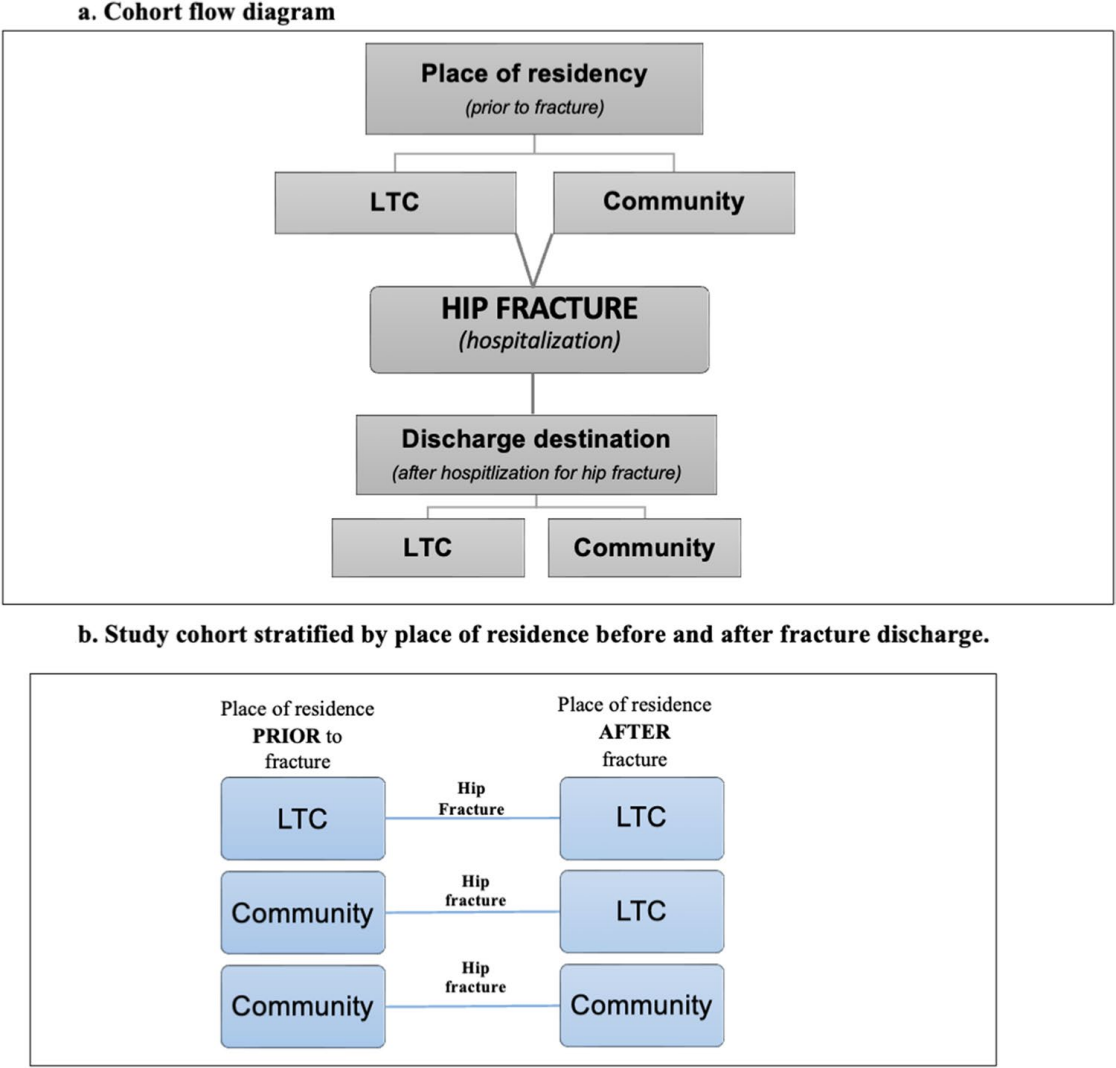
**A** Cohort flow diagram;** b** Study cohort stratified by place of residence before and after fracture discharge

The target outcome was a hip fracture diagnosis within 1 year, stratified by place of residence prior to and after hip fracture (i.e., LTC **OR** community). Multiple LTC admissions or discharges for the same diagnoses were not included. Additional outcome measures included osteoporosis treatment rate among LTC residents at high fracture risk (determined using the FRS and categorized as an FRS score 4 + and osteoporosis treatment rate among LTC residents at each fracture risk level (i.e., 8 risk scores; see Fig. 1).

**Fig. 2:**
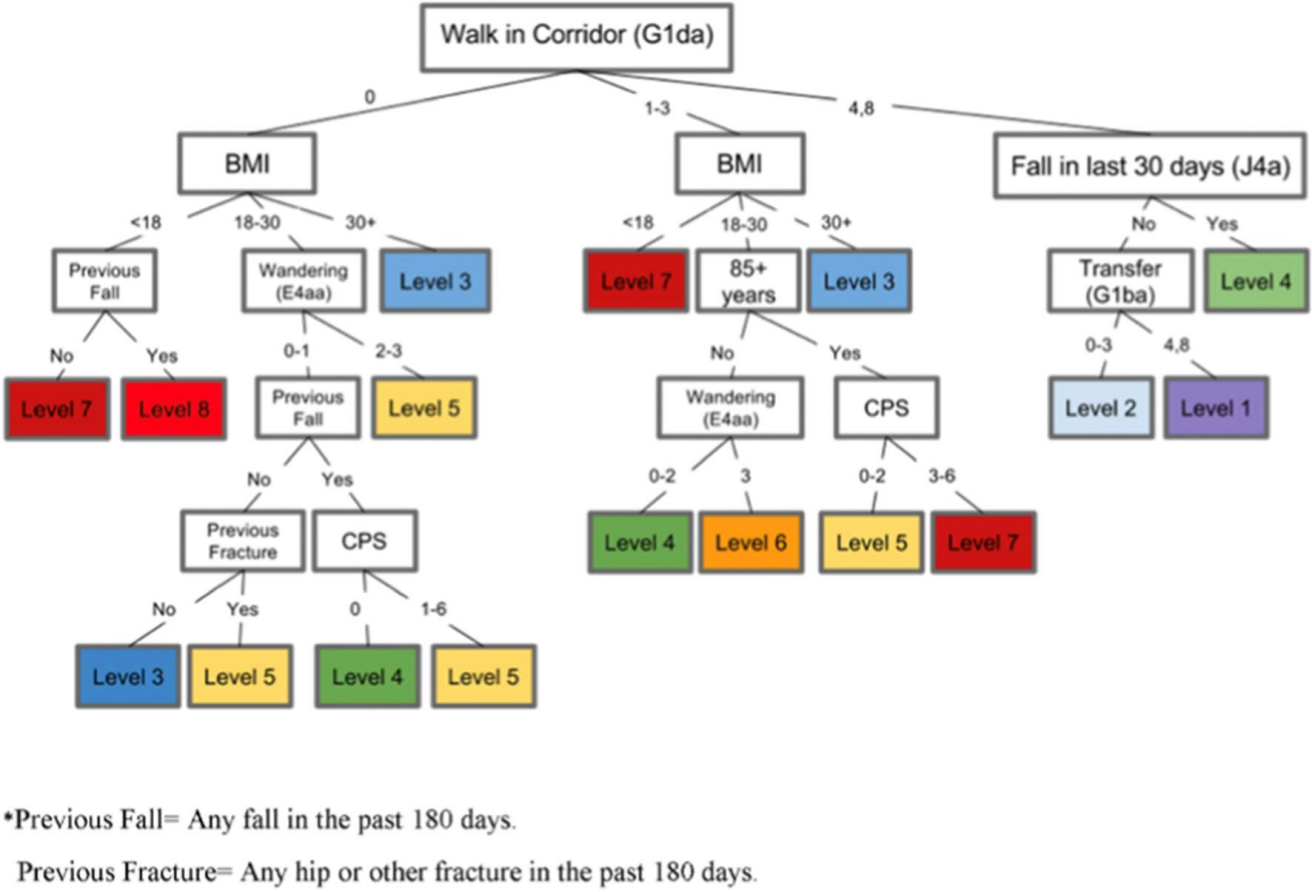
Fracture risk scale (FRS) decision tree analysis [7]

The FRS is the first tool developed and validated to predict hip fracture for LTC residents over a 1-year period using data readily available in RAI MDS 2.0 and LTC-specific risk factors. The FRS was developed using decision tree analysis. Fracture risk assessment progresses through the decision tree until a terminal risk level is determined. The tool includes the following items age greater than 85 years (no/yes), body mass index (BMI) (< 18, 18–30, or > 30 kg/m^2^), walking in corridor (independent, supervision to extensive assistance or total assistance/walking did not occur), wandering (no wandering to infrequent wandering, less than daily wandering, or daily wandering), cognitive performance scale (CPS) (intact cognition, borderline intact or mild impairment, or moderate to very severe impairment), transfer status (how resident moves between surfaces to and from; bed, chair, wheelchair, standing position- categorized as independent to extensive assistance, or total assistance/transfer did not occur), falling status within the past 30 days (no/yes), and previous fractures in the past 180 days (no/yes). FRS consists of 8 risk levels (1 lowest risk, 8 highest risk and categorized as 1–3 risk level = low risk and 4–8 risk level = high risk) (Fig. 2). This categorization is based on observed associations between risk levels and subsequent hip fracture incidence rates in LTC populations [ref development paper]. Individuals with an FRS score of 4 + have a fracture 1-year hip fracture risk > 3% while individuals with a FRS score of 8 have a 1-year fracture risk of 12.6%. Additional details on the FRS have been published elsewhere [7, 22].

### Analysis

Crude hip fracture rates (per 10,000 person-years) were calculated by pre-fracture residence (LTC or community). The numerator included older adults with a hip fracture during the fiscal year of interest (2014/15, 2015/16, 2016/17, 2017/18, 2018/19) and the denominator included all older adults residing in Ontario during that year. Rates were stratified by sex and age groups (66–79, 80 +). Direct Standardization was used to calculate annual age adjusted incident hip fracture rates using the 2017/18 Ontario population. Standardization allows for the comparison of hip fracture rate among different groups over time, while controlling for differences in population size and age distribution. The gamma method [23] was used to calculate confidence intervals for standardized rates. Crude osteoporosis treatment rate (per 100 person-years) was calculated by FRS level (1–8) and stratified by sex and age groups (66–79, 80 +). Additional stratifications by LTC home size (small 1–29 beds; medium 30–99 beds; large $\ge$ 100 beds) and urban/rural status were applied for the primary outcome.

Joinpoint regression analysis was used to examine temporal trends in hip fracture and osteoporosis treatment rates over the study period [24]. This software determines the best-fitting points corresponding to where the rate changes (i.e., increases or decreases) significantly. The analysis starts with zero joinpoints (i.e., the minimum number of joinpoints) and examines whether one or more joinpoints are statistically significant and must be added to the final model. The grid search method was used for fitting the model and the permutation test was used to select the number of joinpoints (number of randomly permuted data sets = 4499) using the Bonferroni correction for multiple testing. The standard error was provided for each data point using the heteroscedastic random error option. The annual percent change (APC) and 95% confidence interval (CI) were determined using the log transformation option. The APC indicates the annual change on a log scale and is scale invariant which allows for comparison across populations or sub-groups. The average annual percent change (AAPC) was computed to summarize the overall trend in rates over the study period. A *p* value of less than 0.05 was taken to indicate a statistically significant effect. All analysis was done using SAS software version 9.4 (SAS Institute Inc) and Joinpoint Regression Program, Version 5.0.2.

## Results

### Hip fracture burden in LTC

Between 2014 and 2018, the number of hip fractures among seniors living in LTC prior to having a hip fracture and discharged back to LTC decreased from 1630 to 1587. During the 5-year observation period, a total of 8241 hip fractures were identified in LTC. At baseline (2014/15), there was a higher number of hip fractures among (1) females (*n* = 1226) compared to males (*n* = 404); (2) those in the 80 + age group (*n* = 1362) compared to the younger age group of 66–79 (*n* = 268) and (3) in urban (*n* = 1406) compared to rural (*n* = 210) setting. Standardization data indicated that overall age-standardized hip fracture rate increased in LTC over the study period from 233.6 per 10,000 person-years in 2014/15 to 315.7 per 10,000 person-years in 2018/19 (Table 1) and was higher among females, individuals 80 + years of age, and LTC residents in rural setting (Table 1).

### Hip fracture burden in the community

At baseline (2014/15), there were 5090 fractures among community-dwelling older adults, which increased slightly in 2018/19 (*n* = 5654 fractures). The number of hip fracture was higher among female (*n* = 3564) compared to males (*n* = 1526), those 80 + years of age (*n* = 3170) compared to those in the 66–79 age group (*n* = 1920), and those living in urban areas (*n* = 4360) compared to rural areas (*n* = 707). The overall age-standardized hip fracture rate decreased from 31.5 per 10,000 person-years in 2014/15 to 26.7 per 10,000 person-years in 2018/19. This pattern was also observed among subgroups (Table 2).

**Table 2:** Age-Standardized Hip fracture rates per 10,000 person-years by fracture residence, sex, and age group

	LTC-LTC				Community-LTC				Community-Community			
	Sex		Age group		Sex		Age group		Sex		Age group	
Year	Female	Male	66–79	80 +	Female	Male	66–79	80 +	Female	Male	66–79	80 +
2014–2015	172.8 (157.8–188.9)	156.9 (138.2–177.4)	129.2 (113.9–144.6)	274.1 (259.7–288.5)	467.7 (425.0–513.5)	319.8 (274.9–369.8)	332.3 (291.6–373.0)	634.0 (600.0–679.9)	43.1 (41.7–44.5)	25.6 (24.3–27.0)	15.1 (14.4–15.8)	92.1 (88.9–95.2)
2015–2016	188.7 (171.9–206.8)	180.0 (159.2–202.9)	150.6 (133.2–168.0)	283.2 (268.7–297.6)	487.2 (440.1–538.0)	280.7 (238.7–328.0)	344.5 (301.5–387.5)	579.6 (542.6–616.6)	38.7 (37.4–40.0)	20.9 (19.8–22.1)	13.8 (13.1–14.4)	78.6 (75.8–81.3)
2016–2017	205.4 (186.1–226.1)	187.8 (165.6–212.2)	167.4 (147.9–186.8)	282.9 (268.2–297.6)	461.4 (412.7–514.3)	329.6 (279.7–385.8)	360.5 (313.5–407.5)	553.2 (516.7–589.7)	36.7 (35.5–37.9)	20.3 (19.2–21.3)	13.9 (13.3–14.5)	72.8 (70.3–75.3)
2017–2018	217.8 (196.5–240.8)	221.0 (195.2–249.2)	184.9 (163.0–206.8)	309.4 (293.3–325.5)	530.2 (473.5–591.9)	357.3 (301.9–420.0)	410.9 (356.9–464.9)	611.6 (572.2–651.0)	33.8 (32.7–34.9)	20.4 (19.4–21.4)	13.8 (13.2–14.4)	66.8 (64.5–69.1)
2018–2019	254.1 (228.8–281.5)	245.5 (216.1–277.7)	211.7 (186.1–237.3)	352.1 (333.5–370.7)	566.9 (503.2–636.4)	407.2 (341.4–482.0)	469.4 (407.4–531.4)	616.0 (574.3–657.7)	33.2 (32.2–34.3)	20.1 (19.2–21.1)	14.9 (14.3–15.5)	61.7 (59.6–63.8)
Absolute change	81.3	88.6	82.5	78.0	99.2	87.4	137.1	− 18.0	− 9.9	− 5.5	− 0.2	− 30.4
Relative change %	47.0%	56.5%	63.8%	28.4%	21.2%	27.3%	41.2%	− 2.8%	− 22.9%	− 21.5%	− 1.3%	− 33.0%

^*^Data is presented as rate per 10,000 person-years (confidence interval). Crude rate per 10,000 is presented for age groups

*“Source: ICES AHRQ Project 2022 0900 819 002”*

### Hip fracture burden in community to LTC group

Between 2014 and 2018, the number of seniors living in the community and discharged to LTC after a hip fracture decreased from 21,885 to 17,233. The number of hip fractures among this cohort decreased from 1171 to 996. At baseline (2014/15), there was a higher number of hip fractures among females (*n* = 890) compared to males (*n* = 281), those in the 80 + age group (*n* = 923) compared to the younger age group of 66–79 (*n* = 248), and in urban (*n* = 1021) compared to rural (*n* = 148) setting. Overall, the age-standardized hip fracture rate increased over the study period, and was higher among females, seniors > 80 years, and seniors living in rural settings (Table 2).

### Comparative trends in hip fracture rate among LTC group, community to LTC group, and sub-groups

**Fig. 3:**
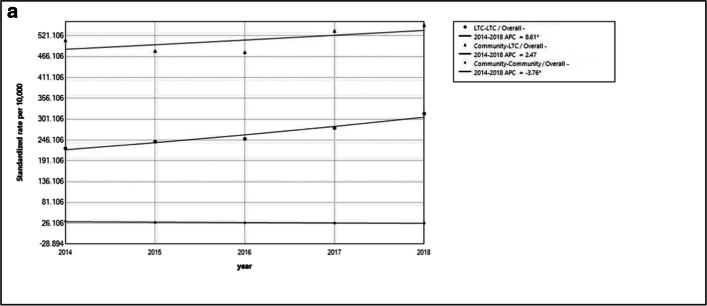

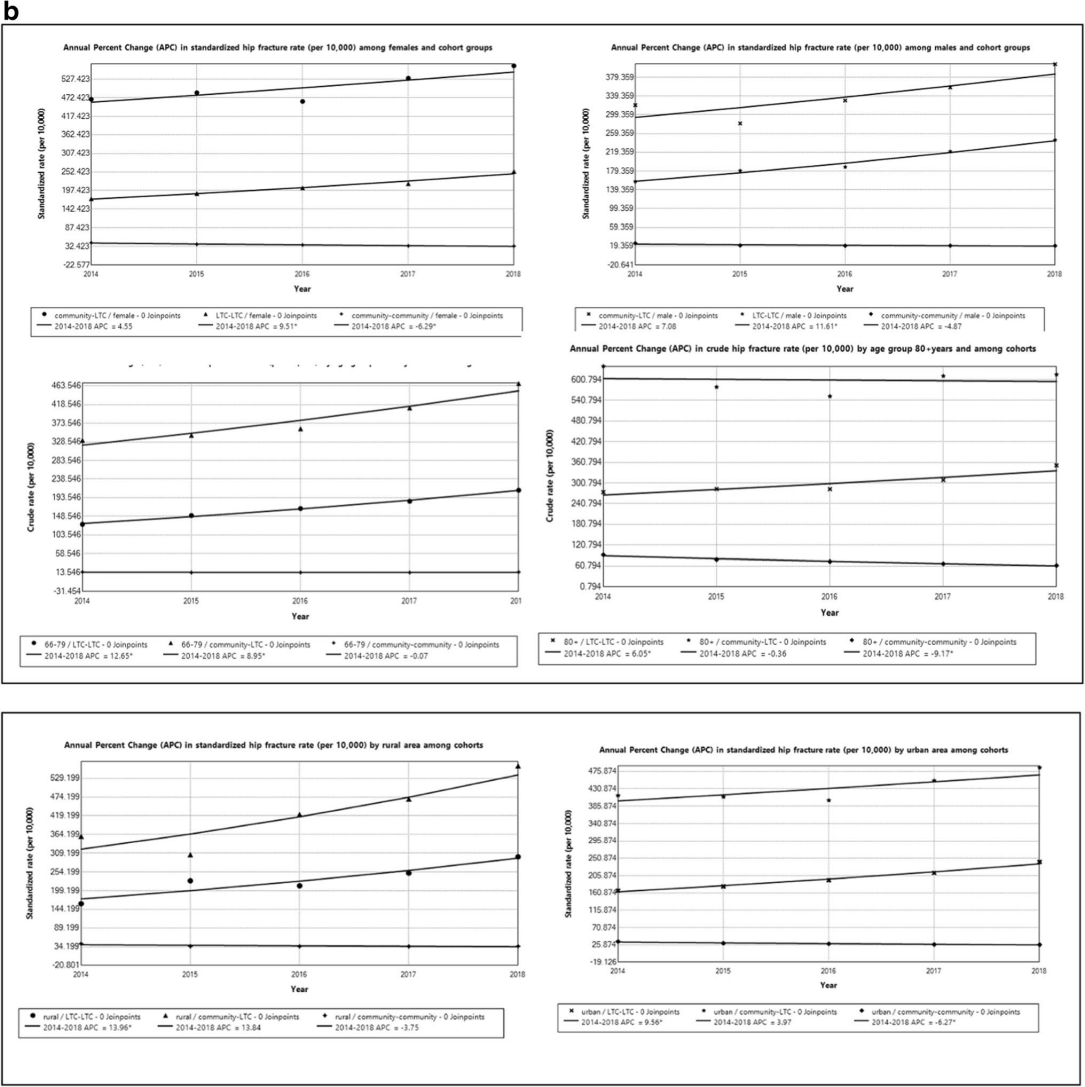
**A**) Overall age-standardized hip fracture rate (per 10,000 person-years) from 2014/15 to 2018/19 among cohorts. **b**) APC across subgroups by sex, age, settings from 2014/15 to 2018/19 among cohorts, APC across subgroups by sex, age, settings

Between 2014 and 2018, the overall annual average percent change (AAPC) increased significantly in LTC (AAPC =  + 8.6; 95% CI 5.0 to 12.3; *p* = 0.004) compared to the community to LTC group (AAPC =  + 2.5 (95% CI − 3.0 to 8.2; *p* = 0.248)) and the community-to-community cohort (AAPC =  − 3.8 (95% CI − 6.7 to − 0.7; *p* = 0.030)) (Table 3). However, the overall hip fracture APC was highest among those in the community to LTC cohort compared to the other cohorts (Fig. 3a).

**Table 3:** Average annual percent change (AAPC) age-standardized hip fracture rate (per 10,000 person-years) 2014/15–2018/19

	LTC-LTC		Community-LTC		Community-Community	
	Annual change, % (95%CI)	*P* value	Annual change, % (95%CI)	*P* value	Annual change, % (95%CI)	*P* value
Overall	8.6* (5.0 to 12.3)	0.004	2.5 (− 3.0 to 8.2)	0.248	− 3.8*(− 6.7 to − 0.7)	0.030
Sex						
Female	9.5* (6.8 to 12.3)	0.001	4.5 (− 0.8 to 10.2)	0.074	− 6.3*(− 8.7 to − 3.8)	0.004
Male	11.6* (8.3 to 15.0)	0.001	7.1 (− 2.2 to 17.3)	0.096	− 4.9(− 11.4 to 2.1)	0.111
Age group						
66–79	12.7* (10.8 to 14.6)	< 0.001	8.9* (4.2 to 13.9)	0.009	− 0.1(− 5.2 to 5.4)	0.971
80 +	6.1* (1.1 to 11.2)	0.030	− 0.4 (− 6.7 to 6.5)	0.874	− 9.2*(− 11.6 to − 6.6)	0.002
Setting						
Rural	14.0* (2.3 to 26.9)	0.031	13.8 (− 0.0 to 29.6)	0.050	− 3.8(− 9.9 to 2.9)	0.164
Urban	9.6* (7.3 to 11.9)	0.001	4.0 (− 1.3 to 9.6)	0.099	− 6.3*(− 9.2 to − 3.2)	0.008

*“Source: ICES AHRQ Project 2022 0900 819 002”*

Hip fracture rate was the highest among females in the community to LTC group and lowest among males in the community-to-community cohort (Fig. 3b). The APC trend increased significantly for both males and females in LTC, there was no significant increase in the APC trend among the sex subgroup in the community to LTC group. In the community-to-community cohort, there was a significant decrease in the APC trend among females but not among males (Table 3b).

Seniors > 80 years in the community to LTC group had the highest hip fracture rates while adults 66–79 years in the community to community had the lowest hip fracture rate over the study period (Fig. 3b). Among the community to LTC, there was a small decrease in the APC between 2014 and 2018. This was not statistically significant (APC =  − 0.4; 95% CI − 6.7 to 6.5; *p* = 0.874). However, the APC increased significantly for adults 66–79 years in community to LTC (APC =  + 8.9*; 95%CI 4.2 to 13.9; *p* = 0.009). A similar significant trend was observed in the LTC cohort among both subgroups (Table 2). Among community-dwelling older adults, a decreasing trend was observed among the age subgroups, with a significant decrease in APC hip fracture rate among seniors > 80 years.

Seniors living in rural settings and in the community to LTC cohort, had the highest hip fracture rate while seniors in the community cohort living in urban areas had the lowest hip fracture rate. The APC increased significantly among seniors living in rural settings among the LTC and community to LTC; however, there was decreasing trend among the community cohort. See the Supplementary information for variations in hip fracture rate among LHINs for both cohorts.

### Osteoporosis treatment rate in LTC among those at high fracture risk (FRS4 +)

At baseline (2014/15), among the 77,189 LTC residents, 14,713 residents were on osteoporosis treatment. Approximately 33,594 LTC residents were at high risk of fracture (FRS score 4 + and up to 12.6% hip fracture yearly incidence rate), of which 7777 were on treatment (23.1%). Among LTC residents at high risk of hip fractures on osteoporosis treatment at baseline (2014/15), they were mostly female (86.3%), older (80 + years) (86.6%), lived in urban settings (90.0%) and were in a large LTC facility size (81.2%).

Between 2014 and 2018, the overall age-standardized treatment rate for LTC residents with an FRS4 + increased from 23.3 to 25.4 per 100 person-years (absolute change 2.1; relative % change 9.0%). Among this cohort, the APC increased for males and females over the study period; however, this trend was only significant for females (*p* = 0.022) (Table 4). The APC increased for LTC residents at high fracture risk (FRS4 +) in both age groups (66–79, 80 + years); however, this was not statistically significant. There was also a non-significant increase in the APC in urban and rural settings. Interestingly, osteoporosis treatment varied by LTC home size. The treatment rate was the highest among large LTC homes and the lowest among small homes. Over the study period, we observed an increase in treatment rate among large and medium LTC home (medium APC =  + 3.1%; 95% CI 1.3 to 5.0; *p* = 0.012); large APC =  + 1.8%; − 1.0 to 4.6; *p* = 0.136). However, there was a significant decrease in osteoporosis treatment for small LTC homes (APC =  − 11.5% (− 20.7 to − 1.1; *p* = 0.039).

**Table 4:** Age-Standardized treatment rate (per 100 person-years) for LTC residents at high fracture risk

	LTC residents FRS 4 +									
		Males		Females		Setting		Facility size		
Year	Overall	66–79	80 +	66–79	80 +	Rural	Urban	Small	Medium	Large
2014–2015	23.3 (22.8–23.9)	8.05 (7.0–9.0)	10.6 (9.9–11.3)	25.6 (24.1–27.1)	29.9 (29.3–30.6)	18.3 (17.0–19.6)	23.9 (23.3–24.5)	18.7 (13.0–26.3)	19.7 (18.6–20.7)	24.2 (23.6–24.8)
2015–2016	23.0 (22.5–23.5)	7.9 (6.9–8.9)	9.8 (9.2–10.5)	27.5 (26.1–29.1)	29.4 (28.8–30.0)	16.7 (15.5–18.0)	23.9 (23.3–24.4)	14.0 (8.8–21.0)	19.6 (18.6–20.7)	23.9 (23.3–24.5)
2016–2017	23.4 (22.9–24.0)	8.3 (7.3–9.3)	9.3 (8.7–10.0)	28.1 (26.6–29.6)	30.2 (29.5–30.8)	18.0 (16.8–19.4)	24.2 (23.7–24.8)	15.6 (10.1–23.1)	20.4 (19.4–21.5)	24.2 (23.7–24.8)
2017–2018	23.6 (23.1–24.1)	8.5 (7.4–9.5)	9.1 (8.5–9.7)	26.3 (24.8–27.8)	30.8 (30.2–31.5)	17.9 (16.6–19.2)	24.3 (23.8–24.9)	11.5 (6.8–18.2)	20.9 (19.8–22.1)	24.3 (23.7–24.9)
2018–2019	25.4 (24.8–25.9)	10.2 (9.1–11.3)	12.0 (11.3–12.7)	26.9 (25.4–28.4)	32.4 (31.7–33.0)	19.0 (17.7–20.4)	26.1 (25.6–26.7)	11.5 (6.8–18.1)	22.1 (21.0–23.4)	26.2 (25.5–26.8)
Absolute change	2.1	2.1	1.4	1.3	2.5	0.7	2.2	− 7.2	2.4	2.0
Relative change %	9.0%	26.7%	13.2%	5.1%	8.4%	3.8%	9.2%	− 38.5%	12.2%	8.3%

^*^Data is presented as rate per 100 person-years (confidence interval). “Source: ICES AHRQ Project 2022 0900 819 002”

### Temporal trends in osteoporosis treatment across fracture risk level in LTC

Over the study period, the osteoporosis treatment rate was the highest among LTC residents with an FRS score of 8 (highest fracture risk) and lowest among LTC residents with an FRS score of 1 (lowest fracture risk). Between 2014 and 2018, there was a significant increase in the treatment rate APC among LTC residents at lowest fracture risk (FRS1) and highest fracture risk (FRS8) (FRS1 APC =  + 5.3; 95% CI 2.0 to 8.7; *p* = 0.015); FRS8 APC =  + 13.2; 95% CI 3.0 to 24.5; *p* = 0.025). Among residents with an FRS score of 3, there was a decrease in the treatment rate APC; however, this was not significant (APC =  − 0.4; 95% CI − 2.8 to 2.0; *p* = 0.623).

Among the male LTC residents 66–79 years, there was an increase in treatment trend over the study period across all FRS levels except for those with an FRS score of 7, where non-significant decrease in the treatment rate was observed (APC =  − 2.9; 95% CI 15.2 to 11.0; *p* = 0.531). Similar trends were observed among male LTC residents > 80 years (see Table 3). Among the 66–79 age group, we saw the greatest increase in treatment rate among males with an FRS score of 4 and among male LTC residents > 80 years we saw the greatest increase in treatment rate among those with an FRS score of 1 or 2 (FRS4 APC = 6.8; 95% CI − 0.7 to 14.8; *p* = 0.063; FRS1 APC = 5.2; 95% CI − 7.8 to 20.0; *p* = 0.309) (Table 5).

**Table 5:** Average annual percent change (AAPC) in age-standardized treatment rate (per 100 person-years) in LTC from 2014/15 to 2018/19

	FRS 4 +		FRS 1		FRS 2		FRS 3		FRS 4		FRS 5		FRS 6		FRS 7		FRS 8	
	Annual change, % (95%CI)	*P* value	Annual change, % (95%CI)	*P* value	Annual change, % (95%CI)	*P* value	Annual change, % (95%CI)	*P* value	Annual change, % (95%CI)	*P* value	Annual change, % (95%CI)	*P* value	Annual change, % (95%CI)	*P* value	Annual change, % (95%CI)	*P* value	Annual change, % (95%CI)	*P* value
Overall	2.0(− 0.7 to 4.7)	0.103	5.3*(2.0 to 8.7)	0.015	3.8(− 0.2 to 8.0)	0.058	− 0.4(− 2.8 to 2.0)	0.623	1.7(− 0.9 to 4.4)	0.125	0.7(− 2.0 to 3.5)	0.458	0.2(− 2.5 to 2.9)	0.865	2.9(− 0.2 to 6.0)	0.059	13.2*(3.0 to 24.5)	0.025
Male																		
66–79	6.0(− 0.6 to 13)	0.064	3.9(− 11.0 to 21.4)	0.487	2.4(− 11.8 to 18.9)	0.649	1.6(− 7.3 to 11.3)	0.619	6.8(− 0.7 to 14.8)	0.063	3.4(− 14.6 to 25.2)	0.620	*	*	− 2.9(− 15.2 to 11.0)	0.531	*	*
80 +	2.3(− 9.3 to 15.4)	0.591	5.2(− 7.8 to 20.0)	0.309	5.2(− 5.4 to 17.0)	0.225	1.7(− 4.8 to 8.7)	0.467	4.4(− 8.3 to 18.9)	0.368	2.8(− 8.9 to 16.1)	0.518	*	*	− 0.3(− 11.8 to 12.9)	0.952	*	*
Female																		
66–79	0.5(− 3.7 to 4.8)	0.759	1.9(− 2.0 to 6.0)	0.229	2.3(− 1.1 to 5.7)	0.121	− 2.8(− 6.0 to 0.6)	0.077	0.5(− 4.1 to 5.2)	0.776	− 2.8(− 9.2 to 4.1)	0.284	*	*	2.5(− 10.7 to 17.8)	0.606	*	*
80 +	2.1(0.0 to 4.3)	0.051	6.2*(4.1 to 8.3)	0.002	4.1*(1.2 to 7.0)	0.020	0.0(− 1.8 to 1.8)	0.969	1.7(− 0.8 to 4.2)	0.118	0.7(− 1.4 to 2.8)	0.385	*	*	3.4*(1.3 to 5.5)	0.014	*	*
Setting																		
Rural	1.5(− 3.1 to 6.3)	0.388																
Urban	2(− 0.5 to 4.6)	0.082																
Facility size																		
Small	− 11.5*(− 20.7 to − 1.1)	0.039																
Medium	3.1*(1.3 to 5.0)	0.012																
Large	1.8(− 1.0 to 4.6)	0.136																

*“Source: ICES AHRQ Project 2022 0900 819 002”*

Between 2014 and 2018, there was an increase in treatment rate among female LTC residents 66–79 years, across all FRS levels except for those with an FRS score of 3 and 5 (APC =  − 2.8; 95% CI − 6.0 to 0.6; *p* = 0.077; APC =  − 2.8; 95% CI − 9.2 to 4.1; *p* = 0.284, respectively). Over the study period, there was also a significant increase in the APC among female LTC residents 80 > years across FRS levels 1, 2, and 7, with the greatest increase observed among individuals with an FRS level of one (FRS1 APC = 6.2; 95%CI 4.1 to 8.3; *p* = 0.002; FRS2 APC = 4.1; 95% CI 1.2 to 7.0; *p* = 0.020; FRS7 APC = 3.4n 95% CI 1.3 to 5.5; *p* = 0.014). The APC for individuals with an FRS score of 3 was stable over the study period (APC = 0.0%; 95% CI − 1.8 to 1.8).

## Discussion

In this population-based study, we report trends in hip fracture and osteoporosis treatment rate in Ontario between 2014 and 2018 among LTC and community-dwelling seniors. Our study examined key similarities and differences in fracture rate among seniors by place of residency prior to fracture and discharge destination (i.e., community or LTC). This distinction (based on place of residence prior to LTC admission) is key as the LTC cohort is older, more frail, more likely to experience cognitive impairment, and are at increased risk of falls and fractures, compared to community-dwelling seniors [25–27]. By differentiating rates of fracture and osteoporosis treatment among individuals living in community and LTC prior to fracture, this study provides valuable insight on specific challenges faced in the LTC sector. It can inform the development of targeted intervention to alleviate the burden of hip fracture in this vulnerable population.

Over the study period, the age-standardized hip fracture rate increased among the LTC and community to LTC cohorts but decreased among seniors in the community. Overall, the hip fracture rate was higher among seniors living in the community and discharged to LTC after hip fracture compared to other groups. Hip fracture rate was higher among females, individuals 80 > years, and those in rural settings among all cohorts. The observed higher hip fracture rate in the community-to-LTC cohort compared to LTC is noteworthy. This cohort may represent the frail older adults at high risk of fracture and living in the community but not receiving appropriate osteoporosis treatment and management. This was observed in a Swedish study examining factors associated with living arrangements before hip fractures [28]. Community-dwelling older adults admitted to a nursing home after a fracture were frailer, more often receiving elder-care services, and were already at higher risk of death before sustaining a hip fracture [28]. Frailty is associated with an increased risk of falls and fractures, so individuals with greater frailty who transition to LTC after their fracture may have been at increased risk of fracture and have worse health outcomes compared to seniors already in LTC [28]. We observed an overall significant decrease in hip fracture rate among the community-to-community cohort. Several factors may contribute to this trend.

Over the past decade, there has been growing awareness and emphasis on fall prevention strategies among older adults in Ontario. Community-based programs such as the Exercise and Falls Prevention Class [29] focusing on strength and balance exercises [30–32], home modifications, and educational tools such as the “You CAN prevent falls” [33], “*The Safe Living Guide—A guide to home safety for seniors*” and the “*Too Fit to Fracture*” [34]. These approaches provide information about fall risk and have shown promising results in reducing the incidence of falls and, consequently, hip fractures [35]. Additional strategies have been implemented to assist healthcare providers in osteoporosis management and fall prevention. For example, the Ontario Osteoporosis Strategy (OOS) developed an osteoporosis and falls assessment tool based on the 2010 Clinical Practice Guidelines for the Diagnosis and Management of Osteoporosis in Canada and the 2015 Clinical Practice Guidelines for the Frail Elderly that can be integrated into the electronic medical records (EMR) with the aim of improving osteoporosis-related care in family practice [36, 37]. These tools help healthcare providers effectively assess patients for falls and osteoporosis ensuring timely and appropriate interventions for improved patient outcomes.

We found the rate of hip fractures to increase in LTC between 2014 and 2018. This trend was different compared to a previously published population-based Canadian study [1], which observed an overall decrease in age-standardized hip fracture rate between 2002 and 2012 (APC − 3.49; 95% CI − 3.97 to − 3.01). This trend was also reported in a 9-year longitudinal American study that evaluated trends in hip fracture rates among 2.6 million newly admitted nursing home residents from 2007 to 2015 [5]. Similar to our study and the Canadian literature, they found a small absolute decrease in the incidence rate of hip fracture between 2007 (3.32/100 person-years) and 2013 (2.92/100 person-years) [5]. However, the rate increased in the year 2015 (3.03/100 person-years) [5]. The change in hip fracture trend over time may be due to several reasons such as temporal differences in the population’s clinical characteristics and vulnerability to fracture. For example, an increase in the proportion of older adults with higher frailty or complex healthcare needs admitted to LTC between 2014 and 2018 could contribute to a higher incidence of hip fractures compared to that of the previous year [5]. However, our study did not have information related to clinical characteristics related to fracture such as comorbidities and frailty, cognitive, and functional status to evaluate this.

The observed increase in hip fracture rate in our study may also be attributed to polypharmacy and deprescription practices. Approximately 61% of Canadians aged 65 and older in LTC homes take 10 or more prescription drugs [38], double the rate among community-dwelling older adults [39, 40]. Polypharmacy has been associated with an increased risk of adverse drug events, falls, and hospitalization [41]. Additionally, higher use of psychotropic drugs, benzodiazepines, antidepressants, and antipsychotics as well as drugs deemed “potentially inappropriate” or “dangerous” is also more commonly observed among LTC residents compared to older adults living at home [42, 43]. This may be due to the patient demographic, as LTC residents are generally older and have more chronic conditions thus require more care and may require more medications. However, psychotropic drugs have been associated with an increased risk of falls while antipsychotics prescribed to older adults with dementia, may be associated with an increased risk of death [42, 43]. The prescription of multiple medications also increases the risk of drug interactions and adverse events. Deprescribing is the planned and systematic practice of identifying and discontinuing medications that may be causing harm or no longer providing benefit [44]. A recent US study [45] found that bisphosphonates were deprescribed in 20% of nursing home residents with dementia. Factors contributing to deprescription included older age, comorbidities, poorer prognosis, swallowing difficulties, less mobility, and drug administration challenges such as nursing shortages. Bisphosphonates are often first line treatment options for osteoporosis prevention as they accumulate in the bone with some antifracture efficacy after cessation of therapy [46–48]. Prescribers may recommend deprescription or “drug-holidays” of bisphosphonates in some cases. However, recent evidence indicates that drug holidays should only be considered in patients at low risk of fracture [49], and recent international guidelines emphasize that osteoporosis treatment is a lifelong consideration, with treatment likely continued in those at highest risk [50].

The observed difference in age-standardized hip fracture rate among subgroups is consistent with a previously published Canadian epidemiological study that found the incidence rate of hip fractures to increase with age, being female, and in rural settings [51]. Several international studies have described similar variability in hip fracture rates among subgroups of LTC residents [5, 25, 51, 52]. For example, a longitudinal retrospective cohort study of care home residents (including residential and nursing homes) in England also found hip fracture rates to be higher among females compared to males and higher among care home residents aged 70–89 years from 2012 to 2019 [52].

Our study found a higher hip fracture rate among residents living in rural compared to urban settings among LTC residents. This may be attributed to limited access to healthcare resources and fewer specialized healthcare facilities in rural areas, resulting in fewer opportunities for osteoporosis treatment and management or delayed access to medical care, and potentially contributing to the higher rates of hip fractures seen in rural areas [53]. Rural areas often not only have LTC facilities with smaller sizes and fewer beds than urban areas but also limited access to formal healthcare services, including home-based care options. This may result in a higher proportion of LTC admissions for individuals needing greater care and support and influence hip fracture rates in this cohort.

In rural settings, several aspects may contribute to an increased risk of falls and fractures among LTC residents. One key factor is the higher prevalence of social isolation and limited social support in these areas [54]. This was observed in an Australian study exploring factors associated with social isolation among LTC residents in rural or remote communities [55]. Visitors to LTC residents in rural regions reported obstacles like time, distance, and transportation issues, which can reduce visitation frequency and leave LTC residents spending extended periods alone [55].

In this study, we found an increase in osteoporosis treatment rate among LTC residents at high risk of fracture (FRS4 +). Between 2014 and 2018, the treatment rate ranged from 23 to 25%; this is similar to a recently published study that reported a 21% treatment rate among LTC residents post-fracture [52]. Other studies have reported treatment rates ranging from 1.5 to 40% among high fracture risk LTC residents [56]. It is well established that there is a large treatment gap among LTC residents at high fracture risk [57–60]. This may be attributed to many reasons including LTC residents rarely being included in clinical trials on osteoporosis treatment making it challenging for clinicians to weigh the likelihood of benefit against potential harms and burden from therapy given the residents preferences, life expectancy and fall risk [61]. Although clinical trials and real-world evidence have shown that osteoporosis therapy is effective for fracture prevention in community-dwelling older adults LTC residents have higher rates of polypharmacy, pill dysphagia, and chronic kidney disease [62–65] which may affect the balance of benefits and risks. Also, since the average life expectancy of older adults entering LTC is 2 years [65] pharmacologic therapy should be provided to patients at high risk of fracture (FRS4 +) with sufficient time to benefit from treatment [66].

Globally, there are limited national guidelines on recommendations for osteoporosis management in LTC with only three published from Canada, the United States, and Australia providing recommendations on osteoporosis treatment and fall injury prevention in the LTC setting [67–69]. The Canadian guidelines were developed in 2015 and provide recommendations for osteoporosis management stratified by high and low fracture-risk residents [68]. The American guidelines was last updated in 2009, so it does not include more recently approved treatment options (e.g., denosumab) although treatments that are no longer widely used (e.g., raloxifene and calcitonin) are included [67]. More recently, the Australian guideline published in 2020, provide evidence-based recommendation on osteoporosis management [69]. For example, oral bisphosphonates are not recommended as first-line treatment, due to the complexities of administration for frail LTC residents (e.g., swallowing difficulty, given on an empty stomach and waiting 30 min prior to breakfast and sitting upright).

Currently, there is no international consensus of optimal fracture risk assessment tools in the LTC setting. Two screening tools (FRAiL and FRS model) have been developed to identify LTC residents at high fracture risk and who are most likely to benefit from treatment; however, these tools have not been extensively studied [7, 70]. Although there is no international consensus for fracture risk assessments in LTC, both of these tools utilize MDS-RAI data and take into consideration falls and fracture risk factors for frail elderly individuals, as such, are embedded in interRAI assessment system. Additionally, in Canada the FRS model has been validated to predict 1-year hip fracture risk [7] whereas in the United States, the FRAiL model [70] has been validated to predict a 2-year risk of hip fracture in LTC. Nevertheless, the process of determining fracture risk and life expectancy and conveying the risks and benefits of initiating osteoporosis therapy in the context of a patient’s health trajectory and goals of care is essential for fracture prevention in LTC.

Strengths of this study include the use of population-based data in Ontario, Canada over a five-year period (2014/15–2018/19) that limits selection bias. A standardized approach was used to identify outcomes from health administrative data obtained from the ICES data repository; hip fractures were identified using the PHAC algorithm and ICD-10 codes, osteoporosis drugs were identified using DIN’s; and fracture risk was determined using the FRS tool. Although health administrative data is reliable in identifying hip fractures, there were sociodemographic (BMI, education, etc.), lifestyle variables (such as smoking, diet), or medical conditions (such as frailty, sarcopenia, falls) or mortality that could be involved in changing the risk of hip fractures. Second, analysis was not adjusted for the date of death within the fiscal year. Third, the osteoporosis treatment rate was determined across FRS levels, although the FRS was developed in 2017, it was not available in the RAI-MDS2.0 until 2019. Implementation strategies have been implemented since. However, the data in this study does not reflect the uptake of the FRS and as such, we cannot comment on the use of the FRS and fracture risk and osteoporosis treatment.

## Conclusion

In summary, rates of hip fracture have increased over the past 5 years among LTC residents and community dwelling older adults, which may precipitate admission to LTC. However, hip fracture rates among community-dwelling adults have decreased. Hip fracture and osteoporosis treatment rate was varied among males/females, age groups, and urban/rural settings and LTC home sizes. Residents in LTC are at very high risk for fracture and need individualized fracture prevention care plans based on their goals of care and life expectancy.

## Supplementary Information

Below is the link to the electronic supplementary material.

Supplementary file1 (DOCX 35 KB)

## Data Availability

The data used in this study were provided by the Institute for Clinical Evaluative Sciences (ICES). Access to these data is restricted and requires approval from the relevant data access committees at ICES. The data are not publicly available due to privacy and confidentiality concerns. Researchers who meet the criteria for access to deidentified data can request access from ICES at www.ices.on.ca.
