# Supplementary material for: Hip fracture rate and osteoporosis treatment in Ontario: A population-based retrospective cohort study
Source: Arch Osteoporos. 2024 Jun 25;19(1):53. doi: 10.1007/s11657-024-01402-6 (PMC11199290; doi:10.1007/s11657-024-01402-6)
Supplement: Supplementary file 1 — Supplementary file1 (DOCX 35 KB) [file 11657_2024_1402_MOESM1_ESM.docx]

**Supplementary data**

**Appendix 1**

**Table1. List of databases**

| **Database** | **Data** |
| --- | --- |
| **Discharge Abstract Database (DAD)** | Compiled by Canadian Institute for Health Information (CIHI) contains information for all admissions to acute care hospitals, rehab, chronic, and day surgery institutions in Ontario. Contains a record of each discharge from an Ontario hospital and was used to identify who had a fracture treated on an inpatient basis. |
| **National Ambulatory Care Reporting System (NACRS)** | Contains information for all patient visits made to hospital- and community-based ambulatory care centres (emergency departments, day surgery units, hemodialysis units, and cancer care clinics). NACRS records were linked with other data sources (DAD, OMHRS) to identify transitions to other care settings, such as inpatient acute care or psychiatric care. Contains a record of each visit to an Emergency Department, and each same day surgery event. Used to identify people who had a fracture treated in an ED or on a same day surgery basis. |
| **Ontario Health Insurance Plan Claims Database (OHIP)** | Contains information on inpatient and outpatient services provided to Ontario residents eligible for the province’s publicly funded health insurance system. Contains records of physician claims. Used to identify fractures treated in a physician's office. |
| **Same Day Surgery Database (SDS)** | Contains information for all patient visits made to day surgery institutions in Ontario. Contains a record of each same day surgery event. Used to identify people who had a fracture treated on a same day surgery basis. |
| **Registered Persons Database files (RPDB)** | Provides basic demographic information (age, sex, location of residence (postal code), date of birth, and date of death for deceased individuals) for Ontario residents. |
| **National Rehabilitation Reporting System (NRS)** | Contains information on rehabilitation admissions following fractures. |
| **Continuing Care Reporting System (for Chronic Care) (CCRS)**  **CCRS- Long Term Care (CCRS-LTC)** | Contains information for individuals receiving facility-based continuing care (i.e., extended, auxiliary, or complex chronic care) in Ontario hospitals and residential care providing 24-hour nursing services (i.e. nursing home). Clinical assessment data (on the physical, functional, cognitive, and social domains of health) is ascertained using the Resident Assessment Instrument Minimum Data Set (RAI-MDS) v2.0. Contains information on chronic care following fractures.  Contains information for individuals in long-term care and includes clinical assessment data using the RAI-MDS V2.0. |
| **Client Agency Program Enrolment (CAPE)** | Registry of patients who have been rostered to receive care from physicians in Ontario and includes the period in which a patient was rostered to a specific physician. Identifies people who are enrolled in primary care groups (used to create the indicator denominators). |
| **Yearly Ontario Population estimates and projections (POP)** | Contains intercensal and postcensal estimates of the Ontario population by sex, age, and geographic areas. All estimates are of the population on July 1 of the given year. Datasets containing the 2014-2018 populations of each LHIN, by age and sex. The 2018 and 2019 population files contain projected population estimates. |
| **Ontario Drug Benefit Claims (ODB)** | Contains prescription medication claims for those covered under the provincial drug program, including adults $\geq$ 65 years, nursing home residents, patients receiving services under the Ontario Home Care program, those receiving social assistance, and residents eligible for specialized drug programs. Main data elements include drug identifier, quantity, # days supplied, date dispensed, cost, and patient, pharmacy, and physician identifiers. Contains records of osteoporosis and non-osteoporosis medication prescriptions. |
| **Master Numbering System (MNS)** | Contains general institution number and location information for all health care institutions operating in Ontario since April 1970. Was used to differentiate between chronic care and long-term care residents. |
| **Drug Identification Number Database (DIN)** | Includes Drug Identification Numbers used in Canada from 1990. |

**Appendix 2**

**Table2. Coding algorithm for hip fracture identification**

| **Hip fracture codes** | **Database** | **Details** |
| --- | --- | --- |
| International Classification of Diseases 10th Revision, Canadian version (ICD-10 CA)  S72.0, S72.1, S72.2 | DAD | Hip fracture diagnosis |
| Canadian Classification of Health Interventions (CCI)  1VA73, 1VC53, 1VA74, 1VA53, 1VA80,1VC74 | DAD | CCI codes on hospital discharge abstract |
| Ontario Health Insurance Plan Claims Database (OHIP)  F100, F101, F096, R440, or R439 | OHIP | OHIP Fee codes billed during hospitalization |

**Appendix 3**

**Age-standardized hip fracture rate by LHIN**

| **Table3. Age standardized hip fracture rate (per 10,000 person-years) among the LTC-LTC cohort** | | | | | | | |
| --- | --- | --- | --- | --- | --- | --- | --- |
|  | 2014 | 2015 | 2016 | 2017 | 2018 |  |  |
| LHINS | Rate (SE) | Rate (SE) | Rate (SE) | Rate (SE) | Rate (SE) | Absolute change | Relative change |
| Erie St. Clair | 188.59(26.59) | 225.52(31.94) | 235.38(34.39) | 259.27(41.08) | 227.56(38.81) | 38.97 | 20.66 |
| South West | 170.01(20.31) | 192.03(20.95) | 254.75(28.34) | 200.73(25.51) | 292.18(35.98) | 122.17 | 71.86 |
| Waterloo Wellington | 133.4(21.93) | 204.31(33.35) | 160.18(27.79) | 217.09(36.86) | 314.86(50.04) | 181.46 | 136.03 |
| Hamilton Niagara Haldimand Brant | 161.29(16.74) | 215.12(21.76) | 170.69(19) | 210.28(22.83) | 246.05(28.84) | 84.76 | 52.55 |
| Central West | 169.14(30.04) | 142.5(24.51) | 210.33(39.23) | 213.07(40.68) | 238.23(44.95) | 69.09 | 40.85 |
| Mississauga Halton | 143.13(24.58) | 104.94(21.64) | 184.68(33.42) | 160.41(31.94) | 241.01(46.88) | 97.88 | 68.39 |
| Toronto Central | 135.88(20.42) | 90.95(13.96) | 173.82(27.61) | 208.21(31.02) | 244.48(35.17) | 108.60 | 79.92 |
| Central | 132.13(17.24) | 163.21(21.72) | 160.63(20.88) | 144.68(20.72) | 222.79(32.37) | 90.66 | 68.61 |
| Central East | 171.9(19.29) | 154.59(18.34) | 194.07(23.05) | 223.06(27.12) | 214.71(26.84) | 42.81 | 24.90 |
| South East | 220.97(32) | 273.18(39.97) | 263.98(40.13) | 316.22(48.43) | 291.65(47.78) | 70.68 | 31.99 |
| Champlain | 185.59(20.88) | 205.58(23.06) | 192.81(23.08) | 214.19(26.38) | 232.66(28.92) | 47.07 | 25.36 |
| North Simcoe Muskoka | 180.03(31.94) | 191.42(33.46) | 200.72(35.81) | 326.59(53.29) | 225.71(41.63) | 45.68 | 25.37 |
| North East | 159.19(22.33) | 237.89(30.35) | 194.71(29.47) | 218.42(31.73) | 252.46(37.71) | 93.27 | 58.59 |
| North West | 247.8(55.41) | 199.47(51.05) | 216.61(54.36) | 366.56(87.36) | 418.61(100.54) | 170.81 | 68.93 |

| **Table4. Age standardized hip fracture rate (per 10,000 person-years) among community-LTC cohort** | | | | | | | |
| --- | --- | --- | --- | --- | --- | --- | --- |
|  | 2014 | 2015 | 2016 | 2017 | 2018 |  |  |
| LHINS | Rate (SE) | Rate (SE) | Rate (SE) | Rate (SE) | Rate (SE) | Absolute change | Relative change |
| Erie St. Clair | 424.83(71.51) | 410.07(68.76) | 414.97(71.47) | 439.26(78.76) | 703.26(131.56) | 278.43 | 65.54 |
| South West | 348.12(43.25) | 340.53(48.12) | 386.77(57.95) | 492.16(72.83) | 384.65(64.4) | 36.53 | 10.49 |
| Waterloo Wellington | 329.68(67.16) | 299.95(67.1) | 264.9(58.75) | 363.34(75.88) | 520.53(108.29) | 190.85 | 57.89 |
| Hamilton Niagara Haldimand Brant | 409.88(43.91) | 391.65(47.63) | 323.52(44.92) | 441.72(57.88) | 420.02(61.76) | 10.14 | 2.47 |
| Central West | 330.89(67.74) | 490.38(86.85) | 431.62(95.82) | 535.88(114.37) | 463.51(119.21) | 132.62 | 40.08 |
| Mississauga Halton | 506.54(79.11) | 343.46(68.87) | 249.64(62.28) | 163.57(50.44) | 367.78(98.29) | -138.76 | -27.39 |
| Toronto Central | 288.99(55.76) | 367.61(73.82) | 345.67(76) | 392.69(82.14) | 343.32(76.16) | 54.33 | 18.80 |
| Central | 467.57(56.57) | 497.46(57.34) | 375.28(52.1) | 494.95(68.37) | 374.57(60.82) | -93.00 | -19.89 |
| Central East | 376.3(47.34) | 347(46.34) | 480.56(62.5) | 463.22(62.84) | 549.95(75.85) | 173.65 | 46.15 |
| South East | 317.56(61.82) | 345.2(70.04) | 369.97(74.34) | 270.48(71.23) | 386.91(97.81) | 69.35 | 21.84 |
| Champlain | 389.99(50.13) | 445.37(55.13) | 476(60.08) | 526.13(68.05) | 637.01(79.9) | 247.02 | 63.34 |
| North Simcoe Muskoka | 617.92(89.1) | 638.68(93.89) | 672.26(105.87) | 689.06(115.75) | 861.85(143.11) | 243.93 | 39.48 |
| North East | 471.15(68.24) | 320.99(56.52) | 423.18(74.55) | 486.92(92.03) | 528.83(95.95) | 57.68 | 12.24 |
| North West | 241.49(95.8) | 170.02(64.66) | 433.16(148.14) | 445.22(140.63) | 482.4(183.39) | 240.91 | 99.76 |

| **Table5. Age standardized hip fracture rate (per 10,000 person-years) among community-community cohort** | | | | | | | |
| --- | --- | --- | --- | --- | --- | --- | --- |
|  | 2014 | 2015 | 2016 | 2017 | 2018 |  |  |
| LHINS | Rate (SE) | Rate (SE) | Rate (SE) | Rate (SE) | Rate (SE) | Absolute change | Relative change |
| Erie St. Clair | 43.02(2.41) | 36.12(2.09) | 38.43(2.05) | 33.4(1.83) | 35.39(1.82) | -7.63 | -17.74 |
| South West | 42.69(1.99) | 39.48(1.8) | 35.37(1.62) | 34.64(1.53) | 30.78(1.38) | -11.91 | -27.90 |
| Waterloo Wellington | 39.37(2.44) | 34.61(2.13) | 34.57(2.03) | 27.54(1.73) | 24.97(1.59) | -14.40 | -36.58 |
| Hamilton Niagara Haldimand Brant | 31.47(1.36) | 23.22(1.1) | 24.78(1.09) | 25.05(1.07) | 22.75(0.97) | -8.72 | -27.71 |
| Central West | 30.25(2.2) | 28.43(2) | 24.96(1.77) | 23.58(1.53) | 24.86(1.6) | -5.39 | -17.82 |
| Mississauga Halton | 27.95(1.7) | 24.03(1.46) | 23.03(1.36) | 18.65(1.18) | 20.36(1.16) | -7.59 | -27.16 |
| Toronto Central | 31.15(1.48) | 26.73(1.31) | 25.3(1.23) | 23.9(1.17) | 23.86(1.14) | -7.29 | -23.40 |
| Central | 32.3(1.37) | 28.24(1.21) | 25.76(1.1) | 25.01(1.03) | 24.68(0.98) | -7.62 | -23.59 |
| Central East | 35.98(1.46) | 35.45(1.37) | 32.08(1.24) | 30.02(1.14) | 27.95(1.07) | -8.03 | -22.32 |
| South East | 41.33(2.62) | 36.62(2.27) | 34.76(2.12) | 34.72(2.03) | 36.32(1.98) | -5.01 | -12.12 |
| Champlain | 42.19(1.82) | 35.1(1.56) | 33.45(1.45) | 31.31(1.33) | 32.33(1.31) | -9.86 | -23.37 |
| North Simcoe Muskoka | 36.12(2.61) | 36.54(2.45) | 31.84(2.17) | 36.85(2.26) | 35.42(2.1) | -0.70 | -1.94 |
| North East | 36.93(2.37) | 32.46(2.09) | 33.85(2.03) | 30.78(1.88) | 32.34(1.85) | -4.59 | -12.43 |
| North West | 46.4(4.5) | 27.7(3.32) | 32.91(3.37) | 29.94(3.06) | 32.79(3.1) | -13.61 | -29.33 |

**Appendix 4**

**Osteoporosis treatment among LTC residents by FRS level**

| **Table6. Overall age-standardized treatment rate (per 100 person-years) by FRS levels** | | | | | |
| --- | --- | --- | --- | --- | --- |
|  | 2014 | 2015 | 2016 | 2017 | 2018 |
| FRS1 | 12.91(0.27) | 13.48(0.28) | 13.83(0.28) | 14.29(0.29) | 16.12(0.3) |
| FRS2 | 19.82(0.32) | 20.24(0.33) | 20.31(0.33) | 20.83(0.33) | 23.47(0.36) |
| FRS3 | 20.52(0.36) | 19.82(0.36) | 19.94(0.36) | 19.4(0.36) | 20.33(0.37) |
| FRS4 | 22.69(0.45) | 22.57(0.45) | 23.11(0.45) | 22.79(0.44) | 24.57(0.46) |
| FRS5 | 24.47(0.52) | 23.99(0.52) | 24.22(0.53) | 23.82(0.54) | 25.5(0.57) |
| FRS6 | 19.62(1.35) | 18.66(1.39) | 19.27(1.34) | 18.89(1.42) | 19.74(1.49) |
| FRS7 | 23.56(0.45) | 23.15(0.44) | 23.53(0.44) | 24.53(0.44) | 26.24(0.46) |
| FRS8 | 24.27(4.09) | 24.6(4.15) | 26.48(4.21) | 30.52(5.57) | 40.27(6.72) |
